# Maximising the impact of qualitative research in feasibility studies for randomised controlled trials: guidance for researchers

**DOI:** 10.1186/s40814-015-0026-y

**Published:** 2015-09-07

**Authors:** Alicia O’Cathain, Pat Hoddinott, Simon Lewin, Kate J. Thomas, Bridget Young, Joy Adamson, Yvonne JFM. Jansen, Nicola Mills, Graham Moore, Jenny L. Donovan

**Affiliations:** 1 Medical Care Research Unit, School of Health and Related Research, University of Sheffield, Regent Street, Sheffield, S1 4DA UK; 2 Primary Care, Nursing Midwifery and Allied Health Professionals Research Unit, University of Stirling, Stirling, FK9 4LA Scotland UK; 3 Global Health Unit, Norwegian Knowledge Centre for the Health Services, Oslo, Norway; 4 Health Systems Research Unit, South African Medical Research Council, Cape Town, South Africa; 5 Institute of Psychology, Health and Society, University of Liverpool, Waterhouse Building, Block B, Brownlow Street, Liverpool, L69 3GL UK; 6 Department of Health Sciences, University of York, Seebohm Rowntree Building, Heslington, York, YO10 5DD UK; 7 Behavioural and Societal Sciences, Work, Health & Care, Schoemakerstraat 97 (Gebouw A), Delft, 2628 VK Netherlands; 8 School of Social and Community Medicine, University of Bristol, Canynge Hall, 39 Whatley Road, Bristol, BS8 2PS UK; 9 Centre for the Development and Evaluation of Complex Interventions for Public Health Improvement, Cardiff University, Cardiff, CF10 3XQ UK

**Keywords:** Randomised controlled trial, Feasibility studies, Pilot studies, Qualitative methods, Guidance

## Abstract

Feasibility studies are increasingly undertaken in preparation for randomised controlled trials in order to explore uncertainties and enable trialists to optimise the intervention or the conduct of the trial. Qualitative research can be used to examine and address key uncertainties prior to a full trial. We present guidance that researchers, research funders and reviewers may wish to consider when assessing or undertaking qualitative research within feasibility studies for randomised controlled trials. The guidance consists of 16 items within five domains: research questions, data collection, analysis, teamwork and reporting. Appropriate and well conducted qualitative research can make an important contribution to feasibility studies for randomised controlled trials. This guidance may help researchers to consider the full range of contributions that qualitative research can make in relation to their particular trial. The guidance may also help researchers and others to reflect on the utility of such qualitative research in practice, so that trial teams can decide when and how best to use these approaches in future studies.

## Introduction

The United Kingdom Medical Research Council (UK MRC) guidance on the development and evaluation of complex interventions recommends an early phase of assessing feasibility prior to a full evaluation [1]. In this feasibility and pilot phase, researchers can identify and address problems which might undermine the acceptability and delivery of the intervention or the conduct of the evaluation. When the outcome evaluation is a randomised controlled trial, this feasibility phase increases the chances of researchers evaluating the optimum intervention using the most appropriate and efficient recruitment practices and trial design. Alternatively, at the feasibility phase, researchers may identify fundamental problems with the intervention or trial conduct and return to the development phase rather than proceed to a full trial. The feasibility phase thus has the potential to ensure that money is not wasted on an expensive trial which produces a null result due to problems with recruitment, retention or delivery of the intervention [2].

Feasibility studies for randomised controlled trials can draw on a range of methods. Some feasibility studies use quantitative methods only. For example, researchers concerned about whether they could recruit to a trial, and whether the intervention was acceptable to health professionals and patients, undertook a pilot trial with outcomes related to recruitment and surveys to measure the acceptability of the intervention [3]. Increasingly, qualitative or mixed methods are being used within feasibility studies for randomised controlled trials. A review of 296 journal articles reporting the use of qualitative research with trials published between 2008 and 2010 identified that 28 % of articles reported qualitative research undertaken prior to the full trial [4]. Qualitative research was not only undertaken with trials of complex interventions but was also used with trials of drugs and devices where researchers recognised the complexity of the patient group receiving the intervention or the complexity of the environment in which the trial was to be undertaken [5]. Yet, there is little guidance available on how to use qualitative methods within feasibility studies for trials. Here, we offer guidance in order to help researchers maximise the opportunities of this endeavour.

### Getting the language right: feasibility studies, pilot studies and pilot trials

Before offering guidance on using qualitative methods at the feasibility phase of a trial, we first need to be clear about the meaning of the term ‘feasibility study’ because the language used to describe the preparatory phase for a trial is inconsistent [6]. These types of studies can be called feasibility or pilot studies, with researchers making no clear distinction between the two when reporting their studies in journal articles [7]. The MRC guidance for developing and evaluating complex interventions describes this as the ‘feasibility and piloting’ stage. The UK funding body, the National Institute for Health Research (NIHR), offers definitions of feasibility and pilot studies, distinguishing between the two [8]. A feasibility study is undertaken to address the question ‘can the planned evaluation be done?’. In contrast, pilot studies are miniature versions of the main study. In the case of a randomised controlled trial, the pilot study is a pilot trial. A feasibility study for a randomised controlled trial does not necessarily involve a pilot randomised controlled trial [1] but may do so, and indeed, some researchers have described their studies as a ‘feasibility study and pilot trial’ in the titles of their journal articles [9]. Other terms may be used to describe a feasibility study for a trial, for example a ‘formative’ study as part of ‘evidence-based planning’ [10] or an exploratory pilot study [11] or a process evaluation with a pilot trial [12]. In this guidance, we use the term ‘feasibility study’ to describe any study that addresses the question of whether the planned evaluation trial can be done regardless of the labels other researchers might use.

### The need for guidance on using qualitative methods in feasibility studies for randomised controlled trials

With the use of qualitative research in feasibility studies for randomised controlled trials becoming increasingly common, guidance on how to do this would be useful to both researchers and those commissioning and reviewing this research. Guidance is available or emerging in areas related to feasibility studies for trials. Guidance exists for undertaking quantitative pilot studies [13, 14], and a Consolidated Standards of Reporting Trials (CONSORT) statement for reporting feasibility studies (rather than undertaking them) is under development [6]. UK MRC guidance has recently been developed for process evaluations undertaken alongside randomised controlled trials [15]. This new guidance recommends that, in most cases, it is useful to use both qualitative and quantitative methods concurrently with a pilot or full trial. It also states that as feasibility studies will usually aim to refine understanding of how the intervention works, and facilitate ongoing adaptation of intervention and evaluation design in preparation for a full trial, qualitative data will likely be of particular value at this stage. However, that guidance does not address in any depth issues specific to the use of qualitative research during the feasibility phase of a trial. There is also guidance for writing proposals for using qualitative research with trials [16] and reporting qualitative research undertaken with trials [5]. However, the feasibility phase of a trial is unique in that it may involve the ongoing adaptation of plans for conducting the trial and of the intervention in preparation for the full trial. Therefore, our guidance complements recent and upcoming guidance by focusing on the role of qualitative research specifically rather than the overall feasibility study and by addressing the iterative nature of research that may occur in feasibility studies for trials.

### The focus of the guidance

This guidance focuses on how to use qualitative research within a feasibility study undertaken prior to a fully randomised controlled trial where the aim is to improve the intervention or trial conduct for the full trial. Appropriate and well-conducted qualitative research can make an important contribution to feasibility studies for randomised controlled trials. The guidance presented here may help researchers to consider the full range of possible contributions that qualitative research can make in relation to their particular trial and reflect on the utility of this research in practice, so that others can decide when and how best to use qualitative research in their studies. Prior to presenting the guidance, we clarify six issues about the scope of the guidance:

- A feasibility study may or may not include a pilot randomised controlled trial.
- The feasibility phase follows the development phase of an intervention, in which qualitative methods may also be used [1]. Although there may be overlap between the development of the intervention and the feasibility phase of the trial, this guidance assumes that an intervention has been developed, but that it might need further modification, including assessment of its practicability in the health care setting.
- Qualitative methods can be used alone or in conjunction with quantitative methods, such as modelling and surveys, in the feasibility phase [1].
- The definition of qualitative research is the explicit use of both qualitative data collection and analysis methods. This is distinguished from trialists’ reflective reports on the problems that they encountered in running a feasibility study and from the use of methods that may draw on qualitative approaches but do not meet our definition. For example, some researchers report using ‘observation’ and ‘field notes’ but show no evidence of qualitative data collection or analysis in their article and do not label these as qualitative research [8]. Reflective practice by trialists and intervention deliverers is important for learning about trial conduct but is not the focus of the guidance presented here.
- The guidance focuses on maximising the opportunities of qualitative research by presenting options, rather than delineating required actions. This is based on the understanding that the strengths of qualitative research are its flexibility and responsiveness to emerging issues from the field.
- The guidance may be used by researchers when writing proposals and undertaking or reporting qualitative research within feasibility studies. If the feasibility study includes a pilot randomised controlled trial, reporting should follow the CONSORT statement that is currently under development [6].

### Processes used to develop the guidance

This guidance is based on the experience of the authors of this paper. The authors came together in a workshop to write this guidance after meeting to discuss a study of how to maximise the value of qualitative research with randomised controlled trials which had been undertaken by two of the authors of this guidance (AOC, KJT) [4, 5]. That study involved undertaking a systematic mapping review of journal articles reporting qualitative research undertaken with randomised controlled trials and interviews with qualitative researchers and trialists; some of these articles are referenced to illustrate points made. Towards the end of this study, the UK MRC Hubs for Trials Methodology Research funded a conference to disseminate the findings from this study and a related 1-day workshop to develop guidance for using qualitative research with trials. The nine workshop members, all of whom are authors of this guidance, were identified for their experience in using qualitative research with trials. One member had also published a review of the use of qualitative research alongside trials of complex interventions [17].

The workshop focused on feasibility studies because these were identified as an underdeveloped aspect of trial methodology. The workshop members put forward items for the guidance, based on their experience and expert knowledge. Discussion took place about the importance of items and the different viewpoints within each item. Draft guidance was produced by AOC after the workshop. Subsequent development of the guidance was undertaken by email correspondence and meetings between sub-groups of the workshop membership. A draft of the guidance was then presented at a meeting of an MRC Methodology Hub for researchers with experience in undertaking qualitative research in feasibility studies for trials. Attendees viewed the guidance as helpful, and further insights emerged from this process, particularly around the analysis domain of the guidance.

## Review

### The guidance

**Fig. 1:**
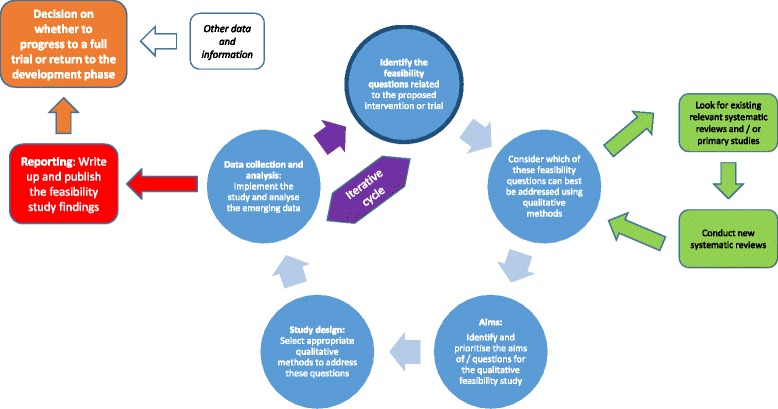
Key steps for qualitative research in a feasibility study for a trial

The guidance is detailed below and summarised in Table 1. The structure follows the stages of a research project from identifying research questions to reporting findings and consists of 16 items within five domains: research questions, data collection, analysis, teamwork and reporting. Although the table presents a neat and linear process, in practice, this research is likely to be messy and iterative, with researchers moving backwards and forwards between steps as insights emerge and the priority of different research questions changes. Figure 1 shows how the guidance meshes with this more dynamic process. We illustrate some of the items in the guidance using case studies of published qualitative research undertaken within feasibility studies for trials. Some items tend not to be visible in publications, particularly those on teamworking, and therefore are not illustrated in these case studies.

**Table 1:** Guidance for using qualitative research in feasibility studies for trials

Aspects of the feasibility study	Issues to consider
1. Research questions	a. When designing the feasibility study, consider the wide range of questions. Then, consider those best addressed by qualitative research.
	b. Prioritise the initial questions by identifying key uncertainties, whilst allowing for the possibility of emergent questions.
	c. Consider the often overlooked questions, such as ‘what is considered to be usual care?’.
2. Design and data collection	a. Consider the range of qualitative methods that might be used to address the key feasibility questions, including dynamic or iterative approaches which allow learning from early qualitative research findings to be implemented before further qualitative research is undertaken as part of the feasibility study.
	b. Select from a range of appropriate qualitative methods to address the feasibility questions and provide a rationale for the choices made; non-participant observation may be an important consideration.
	c. Pay attention to diversity when sampling participants, groups, sites and stage of intervention.
	d. Appreciate the difference between qualitative research and public and patient involvement.
3. Analysis	a. Consider timing of analysis which might be in stages in a dynamic approach.
	b. Many different approaches to analysis can be used, including framework, thematic and grounded theory-informed analysis.
	c. Data can cover a breadth of issues, but the analysis may focus on a few key issues.
4. Teamworking	a. Have a qualitative researcher as part of the feasibility study design team.
	b. Consider relationships between the qualitative researchers and the wider feasibility study team.
	c. Consider who will make changes to the intervention or trial conduct.
5. Reporting	a. Publish feasibility studies where possible because they help other researchers consider the feasibility of similar interventions or trials.
	b. Describe the qualitative analysis and findings in detail.
	c. Be explicit about the learning for a future trial or a similar body of interventions or generic learning for trials.

1. Research questions
  - When designing the feasibility study, consider the full range of questions that could be addressed. Then, consider those best addressed by qualitative research.

Some researchers have produced lists of questions that could be addressed in feasibility studies for trials, focusing on the conduct of the trial and on the intervention [8]. A review of feasibility and pilot trials identified the range of questions *actually* addressed in a subset of feasibility studies that included a randomised controlled trial, [18] although it was not clear which questions were actually addressed by qualitative research. Other researchers have identified frameworks or typologies of questions for feasibility studies. For example, a description of feasibility studies for cancer prevention in the USA identified a typology of the questions addressed and some of the methodologies used [19]. Qualitative research was identified as useful for issues concerning acceptability, implementation, practicality and expansion (in terms of understanding use of a known intervention in a different sub-group). There is also a framework for the work undertaken by qualitative research with trials [4]. Using the latter framework, we drew on the literature cited here and our own experience of feasibility studies to identify the range of issues qualitative research can address in a feasibility study for a trial (Table 2). Although not noted explicitly in Table 2, the context in which the intervention is delivered is relevant to a large number of the questions identified in Table 2 and should be considered during a feasibility study as well as in the full trial [15]. The important role of context within complex intervention trials was highlighted in a recent study which found that contextual threats to trial conduct were often subtle, idiosyncratic and complex [20], and therefore best explored using qualitative research.

**Table 2:** Questions that qualitative research^a^ can address in a feasibility study for a randomised controlled trial

Category of question	Sub-category	Examples of possible questions
Intervention content and delivery	Intervention development	To what extent does the planned intervention need to be refined or adapted to make it more acceptable to users or more relevant or useful to the specific context in which it is delivered?
	Intervention components	Consider the different aspects of the intervention and which are fixed and flexible. The intervention may be different in practice from the planned intervention and may need to be documented so it can be delivered consistently in the full trial.
	Mechanisms of action	How might the intervention be working? How might it produce the outcomes important to the trial? Data collected to address these questions may be interpreted in relation to the theory upon which the intervention is based or may help to develop new theory.
	Perceived value, benefits, harms or unintended consequences of the intervention	What value do service providers and intervention users place on the intervention and the outcomes it plans to deliver? What benefits and harms do they feel they have experienced from the intervention so that these can be measured in the full trial?
	Acceptability of intervention in principle	Are service users or health care providers unhappy with any aspect of the content or delivery of the intervention?
	Feasibility and acceptability of intervention in practice	What are service users or health care providers’ views of the implementation of the intervention? Has implementation varied by setting? Are there any important intervention-context interactions? Should implementation be tailored by setting?
	Fidelity, reach and dose of intervention	Is the right amount of the intervention getting to the right recipients in the right way? Do those delivering the intervention and/or receiving it adhere to the planned intervention? If not, what are the reasons for this? What are the limits of acceptable tailoring of the intervention?
Trial design, conduct and processes	Recruitment and retention	How do the planned recruitment practices work in the field? Do recruitment practices need to be improved to increase recruitment rates and levels of informed consent? If so, how? Are the trial participants willing to be randomised? Are clinicians willing to recruit patients, or are they uncomfortable? Are there ways in which trial procedures could be improved to increase retention rates?
	Diversity of participants	Are the planned recruitment practices likely to result in recruitment of the desired range of participants for the trial? If not, how might recruitment practices be improved?
	Trial participation	How is the planned trial communication implemented by recruiters and received by participants? How can trial communication be improved to ensure recruiters understand patients’ views about participating in the trial?
	Acceptability of the trial in principle	Is the trial design acceptable to patients, recruiters and service providers in principle?
	Acceptability of the trial in practice	Is the trial design acceptable to patients, recruiters and service providers in practice, or are there ways in which participants try to alter the procedures?
	Ethical conduct	Are the informed consent procedures appropriate and acceptable to likely trial participants?
	Adaptation of trial conduct to local context	Will the planned trial procedures allow the trial to operate effectively in the proposed context? Do any changes need to be made to these procedures?
	Impact of trial on staff, researchers, participants and the health system	Does this trial have any unanticipated negative impacts on recruiters, participants, other stakeholders and the health system? How can these impacts be minimised (e.g. workload involved in recruitment, numbers of measures undertaken)?
	Patient and public involvement	How is patient and public involvement best achieved in the trial?
Outcomes	Breadth and selection of outcomes	Are outcomes important to service users selected for measurement in the full trial—both primary and secondary? Do some trial participants feel that they have experienced or noticed improvements in some outcomes that need to be included in the full trial?
Measures	Accuracy of measures	Are the process and outcome measures valid for this participant group?
	Completion of measures	Can completion rates of measures be improved?
	Development of measures	If validated measures do not exist for all the outcomes to be measured in the full trial, can they be developed in preparation for the trial?

^a^Mixed methods research could also be used

(b) Prioritise the questions for the qualitative research by identifying key uncertainties.

Many questions can be addressed in a feasibility study, but resource limitations require that these are prioritised. The whole team will need to identify the key uncertainties that the feasibility study should address. Thereafter, a search of the evidence base for systematic reviews (including mixed reviews based on both qualitative and quantitative researches) relevant to these uncertainties may yield useful insights. Where no systematic reviews exist, and there is no resource to undertake them, studies of similar interventions or similar types of trials may be helpful. Questions on which there is currently little or no existing evidence can then be prioritised for new primary qualitative research.

(c) Consider often overlooked questions.

Researchers commonly use qualitative research to address the acceptability and feasibility of the intervention [10, 21–24] or its perceived benefits [11, 22]. During our workshop, we identified four important questions that can be overlooked and are worth considering:

(i) How do the intervention components and delivery processes work in the real world?

Guidance for process evaluations recommends developing a logic model or explanatory model of the intervention [15]. This logic model includes the intervention components and pathways to delivering desired outcomes. However, even if trialists, intervention deliverers, patients and the public, and qualitative researchers have been involved in developing this logic model, some aspects of the intervention in practice may be hidden or not understood, and these hidden aspects may be the key to delivering outcomes. For example, intervention deliverers may adapt the intervention in unanticipated ways in order to deliver it in their local context. Qualitative research, including non-participant observation and interviews with intervention deliverers and recipients, may be helpful in identifying how and why they have done this. This may facilitate replication of the intervention in the subsequent trial or rollout and also raise questions about the most appropriate trial design required. In addition, it may offer insights into which aspects of the intervention should be fixed or flexible in the full trial [25] and how the intervention needs to be tailored to different contexts. The wider context in which the trial operates may also affect the implementation of the intervention, the control or the trial, for example staff shortages, media scares or the economic climate. Intervention vignettes can be a helpful tool in qualitative interviews to talk potential participants through each step of an intervention in a concrete way [26].

(ii) How does the choice of comparator affect the trial?

The focus of qualitative research undertaken with trials tends to be on the intervention, but qualitative research can also help to understand the control. Interventions can be compared with active controls or usual care, and there may be issues to explore regarding the comparability of an active control and the intervention or the extent to which the trial may change usual care [27]. Such research may help the trial team to consider whether there is sufficient difference between the groups being compared in any trial. For instance, the planned intervention may not be that different from usual care in some settings and may need to be enhanced prior to use in the full trial. Differences between the intervention and usual care will have implications for the relative effectiveness of the intervention and the transferability of the trial findings to other contexts.

Understanding usual care is also important because it represents a key feature of the context in which the new intervention will be implemented. Where a new intervention represents a fundamental change from usual practice, one would perhaps expect to encounter greater challenges in implementation and would need to pay more attention to the resources and structures required to achieve change compared to where the intervention represents a more incremental change.

(iii) To what degree does equipoise exist?

Key stakeholders may not be in equipoise around the intervention [28]. These stakeholders include the trial designers, recruiters, patient and public representatives and participants, as well as health care staff who are not directly involved in the trial but will use the evidence produced by it. A lack of equipoise amongst stakeholders may lead to poor recruitment practices, low recruitment rates or a lack of utility of the evidence in the real world [29]. Consideration of the question of equipoise at the feasibility phase can offer opportunities to address this, for example through education, increasing awareness and enabling open discussion of the issues, or highlight the option of not progressing to an expensive full trial [30, 31]. This has been highlighted as a particular problem for behavioural intervention trials, with recommendations to explore this issue at the pilot stage of a trial [32].

2. Design and data collection
  - Consider the range of qualitative methods that might be used to address the key feasibility questions, including dynamic or iterative approaches which allow learning from early qualitative research findings to be implemented before further qualitative research is undertaken as part of the feasibility study.

When undertaking qualitative research in feasibility studies for trials, it is common for researchers to undertake a cross-sectional interview study with intervention deliverers and recipients and not to specify explicitly an approach or design [12, 21, 22, 24]. Although sometimes it may be important to mirror closely the expected approach of the planned full trial in terms of recruitment practices, it may be helpful for the research team to take a flexible approach to the qualitative research. The team may make changes during the feasibility study itself, based on findings from the qualitative research, and then assess the impact of these changes [33]. This is sometimes called a ‘dynamic approach’. Such changes could include taking action to modify the pilot trial conduct, as well as working with intervention stakeholders to feedback and resolve difficulties in implementing the intervention. Further qualitative research can then be undertaken to inform further improvements throughout the feasibility study. This can help to optimise trial conduct or an intervention rather than simply identify problems with it. Case study 1 describes an example of this dynamic approach to data collection [33].

**Table:** 

**Case study 1:** Donovan and colleagues [33] undertook qualitative research within a feasibility study for a trial of prostate testing for cancer and treatment.
*Research question:* The authors are explicit in the introduction of the paper that the most important uncertainty for the full trial was whether participants would agree to randomisation. Therefore exploring this issue, and ways of improving recruitment, was key to decision making about the feasibility of a full trial.
*Design and data collection:* The qualitative research was a combination of indepth interviews with patients who had undergone the recruitment process and audiotape recordings of recruitment appointments with follow up interviews with recruiters. The data collection and analysis was ‘dynamic’ in that initial qualitative findings were acted on during the feasibility study and further qualitative research undertaken to check if improvements had occurred. The qualitative research showed that recruiters had difficulty discussing equipoise and presenting treatments equally. These findings were summarised and fed back to recruiters in training sessions. Changes were also made to the content and presentation of information in response to findings that patients misinterpreted the language used in the original trial information. Recruitment rates for the pilot trial were monitored over time, showing that they increased as these changes were made.
*Analysis:* Methods of constant comparison were used and references are given.
*Reporting:* The qualitative findings are reported in detail including quotes. The effect of the qualitative research on the full trial is clear in the abstract and the body of the paper. The recruitment rate increased during the pilot trial and three armed trial was identified as feasible.

Other approaches suitable for feasibility studies include iterative ‘rapid ethnographic assessment’ which has been used to adapt and tailor interventions to the different contexts in which the trial was planned [34]. This approach applies a range of methods including participant observation, focus groups, interviews and social mapping [34]. Other researchers have used ‘mixed methods formative research’ at the feasibility stage [10] and action research where potential participants and practitioners are actively involved in the research to assess the feasibility of an intervention and to ensure a good intervention-context fit [35, 36]. For instance, a participatory approach informed by the principles of action research was used to design, implement and evaluate the FEeding Support Team (FEST) intervention [35, 36].

A dynamic or iterative approach to qualitative research in a feasibility study, where concurrent changes are made to the intervention or trial conduct, would not be suitable for a full trial where care is taken to protect the experiment. In a fully randomised controlled trial, researchers may be concerned that an excessive volume or intensity of qualitative research may contaminate the experiment by acting as an intervention [37]. Or, they may be concerned about early reporting of findings of the qualitative research detrimentally affecting staff delivering the intervention or the trial [38]. Any risks will depend on the size and type of the trial and the qualitative research and may be far outweighed by the benefits in practice of undertaking the qualitative research throughout the full trial. These concerns are less relevant during the feasibility phase.

(b) Select from a range of appropriate qualitative methods to address the feasibility questions and provide a rationale for the choices made; non-participant observation may be an important consideration.

Researchers need to select from a range of qualitative methods including telephone and face-to-face interviews, focus groups, non-participant observation, paper/audio/video diaries, case notes kept by health professionals and discussions in online chat rooms and social media. Decisions on data collection and analysis methods should depend on the research questions posed and the context in which data will be collected. To date, feasibility studies for trials have often tended to rely solely upon interviews or focus group discussions with participants and intervention deliverers and have not drawn on the wider range of methods available [21–24]. Researchers tend also to use focus groups and may do this because they think they are cheap and quick when in practice, they are challenging to both organise and analyse. Some researchers are explicit about why focus groups are the best approach for their study. For example, in a randomised trial on the use of diaphragms to prevent sexually transmitted infection, the research team conducted 12 focus groups with women before and after they received the intervention to consider its acceptability and feasibility. This data collection approach was justified on the basis that the researchers felt focus groups would generate more open discussion [10]. However, focus groups may be problematic in a feasibility study because they tend towards consensus and can mask dissenting views, with the possibility of premature conceptual closure. It may also be the case that participants who are prepared to talk openly within a group setting may differ from the target population for a trial as, in general, focus groups tend to attract more educated and confident individuals [39].

Non-participant observation, including the use of audio or video recordings of intervention delivery or recruitment sessions, can help to identify implementation constraints at the feasibility phase. Observation has also proved to be very useful when exploring recruitment practices for a full trial [33, 40]. ‘Think aloud’ protocols may also be helpful—for example, in one feasibility study of a technology to deliver behaviour change, the approach was used to allow users to talk about the strengths and weaknesses of the technology as they attempted to use it [41].

(c) Pay attention to diversity when sampling participants, groups, sites and stage of intervention.

All of the different approaches to sampling in qualitative research—such as purposive, key informant and snowballing—are relevant to feasibility studies. A particular challenge for sampling within the feasibility phase is the need to address the wide range of uncertainties about the full trial or the intervention within the resource limitations of the study.

It can be difficult to decide when enough has been learnt about the trial intervention or the conduct of the trial (or when data saturation has occurred) to recommend moving on to the full trial. Researchers will need to make pragmatic decisions on which emerging analysis themes warrant more data collection and where sufficient data are available. In practice, sample sizes for qualitative research in feasibility studies are usually small (typically between 5 and 20 individuals [10, 12, 22–24]). This may be reasonable, given that simulations suggest that 10 users will identify a minimum of 80 % of the problems with the technology during usability testing, and 20 users will identify 95 % of the problems [42]. However, sample size will be dependent on the study; for example, there may be therapist effects to consider and a need to sample a range of patients using different therapists or a range of contexts.

Diversity of sampling is probably more important at the feasibility phase than the number of interviews or focus groups conducted, and some researchers have rightly highlighted as a limitation the lack of diversity in the sampling process for their qualitative feasibility study [20]. Paying attention to the diversity of sampling needed may be important for identifying the wide range of problems likely to be faced by the group/s to which the intervention is directed. Including a diverse range of health professionals and patients (for an individual-level trial) and sites (for a cluster trial) can be beneficial. In individual-level multicentre trials, including more than one centre at the feasibility stage can reduce the chance of refining an intervention or trial that will only work within that single centre. As in other forms of qualitative research, sampling may be very broad at the start of the feasibility study, when there are lots of questions and uncertainty, with later sampling focusing on disconfirming cases to test emerging findings.

(d) Appreciate the difference between qualitative research and public and patient involvement.

In the UK and many other settings, it is considered good practice to have public and patient involvement in health research [43]. This is highly relevant to a feasibility study where patients and the public can contribute to prioritising which key uncertainties to address and are therefore involved at an early stage of the design of the full trial. Indeed, there is guidance available on patient and public involvement in trials, showing how service users can be involved at the feasibility/pilot stage of a trial by being members of the management group, steering committee and research team and by contributing to the design, analysis and reporting of the feasibility study [44]. A potential concern is that some researchers conflate qualitative research and public and patient involvement; this may be more common during a feasibility study if the public or patient involvement group is asked to provide feedback on the intervention. Although patient and public representatives on research teams can provide helpful feedback on the intervention, this does not constitute qualitative research and may not result in sufficiently robust data to inform the appropriate development of the intervention. That is, qualitative research is likely to be necessary in conjunction with any patient and public involvement. Case study 2 describes an example of a qualitative study undertaken with patient involvement [45].

**Table:** 

**Case study 2:** Hind and colleagues [45] use qualitative research to explore the acceptability of computerised cognitive behavioural therapy for the treatment of depression in people with multiple sclerosis. This is undertaken in the context of a wider study which included a pilot randomised controlled trial.
*Research question:* In the introduction of the paper the authors reference previous research which identifies the importance of exploring whether an intervention engages specific target groups, and the importance of understanding the acceptability of computerised cognitive behaviour therapy.
*Design and data collection:* A patient representative was a member of the research team and was involved in the design and conduct of the study. Data collection for the qualitative study consisted of face-to-face semi-structured interviews with 17 patients who had used one of two computerised cognitive behaviour therapy packages. There was also brief weekly written feedback from patients and brief telephone interviews at the start of the intervention to identify immediate problems.
*Analysis:* Framework analysis was used and is referenced. A patient representative participated in the analysis of the data.
*Reporting:* Although not mentioned in the title of the paper, or the abstract, the authors are clear in the introduction that this paper that the qualitative research was undertaken in the same study as a pilot trial. The qualitative findings are described in detail using quotes from participants. The conclusions relate to the intervention - that computerised cognitive behaviour therapy packages would need to be adapted for people with chronic physical disease - but are not explicit about the implications for a full randomised controlled trial.

3. Analysis
  - Consider the timing of analysis, which might be in stages in a dynamic approach.

For many types of qualitative research, it is suggested that data are analysed as they are collected so that the sampling for the next round of data collection benefits from the analysis of these earlier data. If a dynamic approach is applied in a feasibility study, it is important to have available sufficient resources to analyse the data collected early in the study in order to feed findings back to the wider team and allow changes to be made to the intervention and trial conduct prior to the next set of data collection. This can be quite different from using qualitative research in the full trial, where all data might be collected prior to any formal analysis and sharing of findings with the wider team.

(b) Many different approaches to analysis can be used, including framework, thematic and grounded theory-informed analysis.

Many different approaches can be used to analyse qualitative data in the context of a feasibility study, and the approach should be chosen based on the research question and the skills of the research team. Some researchers simply describe the steps they take within their analysis rather than citing a named approach [12]. Other researchers use combinations of known approaches such as framework analysis and grounded theory [36].

(c) Data can cover a breadth of issues, but the analysis may focus on a few key issues.

An important challenge for analysis may be the specificity of the questions that need to be addressed by a qualitative feasibility study, in order to inform trial development. Analysis will need to focus on the questions prioritised at the beginning, or those emerging throughout the feasibility study, from the large amounts of qualitative data generated. The analysis process needs to consider ‘fatal flaws’ that may require tailoring or refining of the intervention or trial conduct, as well as the mechanisms of action for the intervention.

4. Teamworking
  - Have a qualitative researcher as part of feasibility study design team.

Planning the feasibility study needs qualitative expertise to determine what can be done, how long it might take, how it is best done and the resources needed. It is therefore important that an expert in qualitative methods be included in both the planning and delivery teams for the feasibility study.

(b) Consider relationships between the qualitative researchers and the wider feasibility study team.

How the qualitative researchers interact with the wider feasibility study team is an important concern. If study participants view the qualitative researchers as closely aligned with the team delivering the intervention or conducting the pilot trial, then participants may feel less able to offer honest criticisms of the intervention or trial conduct. On the other hand, where qualitative researchers work too independently from the wider team, they may not develop a deep understanding of the needs of the trial and the implications of their findings for the trial.

Qualitative researchers may identify issues that are uncomfortable for the rest of the research team. For example, they may consider that an intervention does not simply need refining but has a fundamental flaw or weakness in the context in which it is being tested. This may be particularly difficult if the intervention developer is part of the team. Indeed, some members of the team may not be in equipoise about the intervention (see earlier); they may have strong prior beliefs about its feasibility, acceptability and effectiveness and be unable to acknowledge any weaknesses. However, without openness to change, the qualitative research is unlikely to reach its potential for impact on the full trial. On the other hand, the wider team may need to challenge the findings of the qualitative research to ensure that any proposed changes are necessary. Qualitative researchers may also identify problems with the trial conduct that the rest of the team do not see as important because, for example, the recruitment statistics are adequate or it is an effort to change plans. There may also be tensions between what the trial design team need and what the qualitative researcher sees as important. For instance, the trial team may want to understand the feasibility of the intervention whilst the qualitative researcher is more interested in understanding mechanisms of action of the intervention. The team will need to discuss these differences as they plan and undertake the research. The only solution to these tensions is open communication between team members throughout the feasibility study.

(c) Consider who will make changes to the intervention or trial conduct.

Qualitative researchers can identify strengths and weaknesses of the intervention or the conduct of the trial. However, they are usually not responsible for redesigning the intervention or trial either during the feasibility study (if a dynamic approach is taken) or at the end of the feasibility study when the full trial is being considered and planned. It is helpful to be explicit about who is responsible for making changes based on the qualitative findings and how and when they will do this.

5. Reporting
  - Publish feasibility studies where possible because they help other researchers to consider the feasibility of similar interventions or trials.

Other researchers can learn from feasibility studies, and where this is likely to be the case, we recommend publishing them in peer-reviewed journal articles. Other researchers might be willing to take forward to full trial an intervention that the original researchers were unable or unwilling to take beyond the feasibility study. Or, other researchers might learn how to develop better interventions or trials within the same field or understand which qualitative methods are most fruitful in different contexts. Publishing what went wrong within a feasibility study can be as helpful as publishing what went right. Explicit description of how decisions were made about which research questions and uncertainties were prioritised may help others to understand how to make these types of decisions in their future feasibility studies.

Researchers may choose to publish the qualitative findings in the same article as the findings from the pilot trial or quantitative study or may publish them separately if there are detailed and different stories to tell. For example, Hoddinott and colleagues published separate articles related to the outcome evaluation and the process evaluation of a feasibility study of a breastfeeding intervention for women in disadvantaged areas [35, 36]. Feasibility studies may generate multiple papers, each of which will need to tell one part of a coherent whole story. Regardless of how many articles are published from a single feasibility study, identifying each one as a feasibility study in the article title will help other researchers to locate them.

(b) Describe the qualitative analysis and findings in detail.

When publishing qualitative research used with trials, researchers sometimes offer very limited description of the qualitative methods, analysis and findings or rely on limited data collection [5, 17]. This ‘qual-lite’ approach limits the credibility of the qualitative research because other researchers and research users cannot assess the quality of the methods and interpretation. This may be due to the word limits of journal articles, especially if a range of quantitative and qualitative methods are reported in the same journal article. Electronic journals allowing longer articles, and the use of supplementary tables, can facilitate the inclusion of both more detail on the methods used and a larger number of illustrative data extracts [12]. Researchers may wish to draw on guidelines for the reporting of qualitative research [46].

(c) Be explicit about the learning for a future trial or a similar body of interventions or generic learning for trials.

Qualitative research in a feasibility study for a trial can identify useful learning for the full trial and for researchers undertaking similar trials or evaluating similar interventions. This makes it important to be explicit about that learning in any report or article. Reporting the impact of the qualitative research on the trial, and potential learning for future trials, in the abstract of any journal article can make it easier for other researchers to learn from the qualitative research findings [12]. Examples of the impact that qualitative research in feasibility studies can have on the full trial include changes in the information given to participants in the full trial [10], recruitment procedures [21, 28], intervention content or delivery [12, 22, 24], trial design [23] or outcome measures to be used [47]. For example, in the ProtecT trial, initial expectations were that only a two-arm trial comparing radical treatments would be possible, but following the qualitative research, an active monitoring arm that was acceptable was developed and included in the main trial [21]. Learning from the qualitative research may be unexpected. For example, the aim of the qualitative research in one feasibility study was to explore the acceptability of the intervention, but in practice, it identified issues about the perceived benefits of the intervention which affected the future trial design [23]. See case study 3 for an example of qualitative research undertaken with a pilot trial where the learning for the full trial is explicitly reported in the published paper [47]

**Table:** 

**Case study 3:** Farquhar and colleagues [47] undertook a qualitative study to explore the feasibility, acceptability and appropriateness of a widely used quality of life instrument. This was undertaken within a pilot trial in preparation for a Phase III trial of a complex intervention for intractable breathlessness in patients with advanced chronic obstructive airways disease.
*Research question:* In the introduction of the paper the authors explain that it was important to explore the feasibility and acceptability of the instrument because it had not been used with this patient group.
*Design and data collection:* The instrument is administered in the context of an interview. Data collection for the qualitative study consisted of the audio-recordings of these interviews. 13 patients in the intervention and control arms of the pilot trial completed the instrument on 3-5 occasions each.
*Analysis:* Framework analysis was used and is referenced.
*Reporting:* Although not mentioned in the title of the paper, the authors are clear in the abstract that this qualitative research was undertaken in the context of a Phase II trial in preparation for a Phase III trial. The key words include ‘feasibility studies’. Within the methods section of the paper they state that the pilot trial was published elsewhere and give references so that readers can connect the different components of this study if required. The qualitative findings are described in detail using quotes from participants; they identify the difficulties participants had completing the instrument at different stages of the pilot trial. The authors state clearly in the conclusion section of the paper the implications of this work for both the full trial and for the future development of the instrument. The instrument was rejected for use in the full trial because of the difficulties identified.

.

Once a feasibility study is complete, researchers must make the difficult decision of whether to progress to the full trial or publish why a full trial cannot be undertaken. There is guidance on how to make this decision, which encourages the systematic identification and appraisal of problems and potential solutions and improves the transparency of decision-making processes [48]. Too often, progression criteria are framed almost entirely in quantitative terms and it is unclear the extent to which qualitative data may or not play a direct role in informing the decision on whether to proceed to a full trial. For example, if researchers fall just short of a quantitative criterion, but have a sufficient qualitative understanding of why this happened and how to improve it, then it might be possible to proceed. Related to this, qualitative research may identify potential harms at the feasibility stage; the intervention could be modified to avoid these in the full trial, or a decision could be made not to proceed to a full trial even if progression criteria were met.

## Conclusions

Exploring uncertainties before a full trial is underway can enable trialists to address problems or optimise the intervention or conduct of the trial. We present guidance that researchers, research funders and reviewers may wish to consider when assessing or undertaking qualitative research in feasibility studies. This guidance consists of 16 items framed around five research domains: research questions, data collection, analysis, teamwork and reporting. A strength of the guidance is that it is based on a combination of experiences from both published feasibility studies and researchers from eight universities in three countries. A limitation is that the guidance was not developed using consensus methods. The guidance is not meant as a straitjacket but as a way of helping researchers to reflect on their practice. A useful future exercise would be to develop worked examples of how research teams have used the guidance to plan and undertake their qualitative research within feasibility studies for trials. This would help to highlight the strengths and limitations of the guidance in different contexts. Using qualitative research with trials is still a developing area, and so, we present this guidance as a starting point for others to build on, as understanding of the importance of this vital stage of preparation for randomised controlled trials grows. Researchers may also wish to reflect on the utility of different qualitative methods and approaches within their studies to help other researchers make decisions about their future feasibility studies.

## Abbreviations

MRC: Medical Research Council
NIHR: National Institute for Health Research
UK: United Kingdom
